# Ensemble modeling of the potential distribution of the whale shark in the Atlantic Ocean

**DOI:** 10.1002/ece3.5884

**Published:** 2019-11-28

**Authors:** José C. Báez, Ana Márcia Barbosa, Pedro Pascual, María Lourdes Ramos, Francisco Abascal

**Affiliations:** ^1^ Instituto Español de Oceanografía Centro Oceanográfico de Málaga Fuengirola Málaga Spain; ^2^ Facultad de Ciencias de la Salud Universidad Autónoma de Chile Santiago de Chile Chile; ^3^ Faculdade de Ciências CICGE ‐ Centro de Investigação em Ciências Geo‐Espaciais Observatório Astronómico Prof. Manuel de Barros Universidade do Porto Vila Nova de Gaia Portugal; ^4^ Instituto Español de Oceanografía Centro Oceanográfico de Canarias Santa Cruz de Tenerife Spain

**Keywords:** Chondrichthyes, climatic change, marine species, sharks, species distribution modeling, tropical areas

## Abstract

The whale shark (*Rhincodon typus*) is an endangered marine fish species which can be adversely affected by the fishing activities of the industrial purse seine fleet targeting tropical tuna. Tuna tend to aggregate around all types of floating objects, including whale sharks. We analyzed and modeled the spatial distribution and environmental preferences of whale sharks based on the presence and absence data from fishing observations in the Atlantic Ocean. We used a thorough multialgorithm analysis, based on a new presence–absence dataset, and endeavored to follow the most recent recommendations on best practices in species distribution modeling. First, we selected a subset of relevant variables using a generalized linear model that addressed multicollinearity, statistical errors, and information criteria. We then used the selected variables to build a model ensemble including 19 different algorithms. After eliminating models with insufficient performance, we assessed the potential distribution of whale sharks using the mean of the predictions of the selected models. We also assessed the variance among the predictions of different algorithms, in order to identify areas with the highest model consensus. The results show that several coastal regions and warm shallow currents, such as the Gulf Stream and the Canary and Benguela currents, are the most suitable areas for whale sharks under current environmental conditions. Future environmental projections for the Atlantic Ocean suggest that some of the suitable regions will shift northward, but current concentration areas will continue to be suitable for whale shark, although with less productivity, which could have negative consequences for conservation of the species. We discuss the implications of these predictions for the conservation and management of this charismatic marine species.

## Graphical abstract

We assessed the potential distribution of whale sharks using the mean of the predictions of the selected models. Future environmental projections suggest that some of the suitable regions will shift northward.

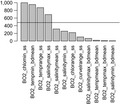

## 1 INTRODUCTION

It is widely accepted that the planet is experiencing a period of rapid global warming (Oreskes, 2004), largely driven by human activity (Keller, 2007), which could adversely affect marine biodiversity and fisheries ecology. Tropical marine ecosystems are especially vulnerable to climate change (Booth, Feary, Kobayashi, Luiz, & Nakamura, 2017; Dell'Apa, Carney, Davenport, & Vernon, 2018). Many studies have predicted that climate change will have strong adverse effects on marine tropical habitats, such as loss of habitat, decreased oxygen concentrations, and increased temperatures and acidification (e.g., Dell'Apa et al., 2018).

In this context, charismatic marine megafauna have been proposed as flagship species to increase public awareness concerning the future of marine ecosystems and the impact of climate change. Such species increase public attention and spark conservationist concern for several reasons related to their role in marine biodiversity (Albert, Luque, & Courchamp, 2018; Zacharies & Roff, 2001).

The whale shark *Rhincodon typus* Smith 1828 is a filter‐feeding elasmobranch and is the largest fish in the world (Chen, Liu, & Joung, 1997; Compagno, 2001). McClenachan, Cooper, Carpenter, and Dulvy (2012) considered the species that appeared in the film “Finding Nemo” to be charismatic species in order to assess their risk of extinction. Using a similar rationale as in their study, the appearance of the whale shark as one of the main characters in the film “Finding Dory,” as well as the public perception of the whale shark, also entitles it to be considered a charismatic species. Such cinematic species are especially likely to gather public and media support for the conservation of marine biodiversity, which makes them valuable research targets (McClenachan et al., 2012).

The whale shark is categorized as endangered in the IUCN red list (Pierce & Norman, 2016). For this reason, and due to its late maturation (around 25 years) and longevity (around 70 years), it is sensitive to mortality by unnatural causes (Escalle et al., 2016). The whale shark can be caught as bycatch by the industrial purse seine fleet targeting tropical tuna (i.e., *Katsuwonus pelamis*,* Thunnus albacares*, and *Thunnus obesus*). Tuna tend to aggregate around all types of floating objects (both natural and artificial), including whale sharks (Gaertner, Ménard, Develter, Ariz, & Delgado De Molina, 2002; Pallarés & Petit, 1998). Despite having very low direct mortality (between 0.93% and 2.53%; Capietto et al., 2014; Escalle et al., 2016), intentional setting on whale sharks is prohibited by most of the Tuna Regional Fishery Management Organizations. Despite its potential detrimental effects, the tuna fishing industry is also the main data source to report whale shark sightings in the open ocean (Sequeira, Mellin, Rowat, Meekan, & Bradshaw, 2012).

Whale sharks migrate over long distances, frequently returning to their area of origin after several years (Robinson, Jaidah, et al., 2017). Thus, whale sharks may perform long‐distance migrations to find highly productive feeding areas (Ramírez‐Macías, Vázquez‐Juárez, Galván‐Magaña, & Munguía‐Vega, 2007; Sequeira, Mellin, Meekan, Sims, & Bradshaw, 2013). However, acoustic telemetry has demonstrated that, although whale sharks have year‐round residency in specific areas, they use a different habitat in the off‐season, swimming deeper and further away from shore, presumably in response to prey distributions (Cagua et al., 2015). A characteristic behavior and distribution of whale sharks is that they aggregate near coasts in shallow areas (Copping, Beukers‐Stewart, McClean, Hancock, & Rees, 2018). There are around 17 key aggregation areas around the world that are dominated by juvenile males (McCoy et al., 2018; McKinney et al., 2017). Peaks in whale shark occurrence appear to happen synchronously in different locations around the world (Sequeira et al., 2013). Thus, in these key areas, the location of aggregation sites could be due to increased prey availability and to a seasonal component (Cárdenas‐Palomo, Herrera‐Silveira, & Reyes, 2010; Kumari & Raman, 2010; Sequeira et al., 2013). For example, in the Azores (eastern Atlantic Ocean), whale sharks mainly aggregate at the end of August and September, although this does not occur every year (Sequeira et al., 2013).

Globally, the whale shark has a wide circumtropical distribution (Pierce & Norman, 2016), although it's specific occurrence areas are not known in detail (Sequeira et al., 2013). Many authors have suggested that this distribution is limited by sea surface temperature (SST), because this species has rarely been observed in waters with SSTs under 21°C (e.g., Pierce & Norman, 2016). Previous modeling studies have been conducted using the fishing logbooks of 65 industrial vessels under different flags operating in the eastern Atlantic Ocean and the western Indian Ocean. They found that the main hotspots of fishery and whale shark interactions were in the ocean area close to the coastal regions between Gabon and Angola in the Atlantic Ocean from April to September, and in the Mozambique Channel in the Indian Ocean between April and May (Capietto et al., 2014; Escalle et al., 2016). There is increasing concern on the effect of warming season whale shark (see Sequeira et al., 2013, and references therein).

Models based on species occurrence data and environmental variables are essential tools to gain insight on species distributions and obtain crucial knowledge for biodiversity conservation and management (see Robinson, Nelson, Costello, Sutherland, & Lundquist, 2017, for a review focused on marine species). The main aim of the present study was to contribute to the knowledge on possible effects of climate change on the distribution and habitat of the whale shark. This study is part of an emerging effort to understand the impact of climatic change on charismatic and vulnerable marine species. We endeavored to follow the most recent best‐practice recommendations for correlative modeling (Araújo et al., 2019; Sofaer et al., 2019), particularly in marine environments (Robinson, Nelson, et al., 2017). Namely, (a) we used actual observation data including both presence and surveyed absence localities (i.e., places where observers were active and did not detect the target species)—which is an uncommon asset in species distribution and niche modeling studies, particularly those targeting marine species; (b) We used the same spatial resolution for species occurrence and environmental data, filtering out any records with insufficient precision; (c) We employed a systematic procedure for selecting relevant predictor variables, avoiding correlated or noninformative variables and backing up their ecological meaningfulness with the literature; (d) We computed and displayed the predictions of a range of different modeling algorithms, and addressed model‐based uncertainty by assessing prediction variance; (e) We cross‐evaluated model predictions over a range of random test samples using both threshold‐dependent and threshold‐independent metrics; we selected models based on their predictive performance on the test samples; and we built the final models using the complete (training plus test) dataset.

## 2 DATA AND METHODS

### 2.1 Predictor variables

We used the R package *sdmpredictors* version 0.2.8 to access potential predictor variables for which both current and future projections were available for the study area. We chose the Bio‐ORACLE version 2.0 dataset (Assis et al., 2018) and the coupled atmosphere‐ocean general circulation models (AOGCM), which provided the most complete set of variables. These variables represent temperature, salinity, chlorophyll concentration, and current velocity, among other factors, both on the sea surface and in deeper waters (Table 1). Present values refer to the period between 2000 and 2014. Future projections for the AOGCMs address the periods 2040–2050 and 2090–2100 under different greenhouse gas concentration scenarios based on different representative concentration pathways (RCP). We used the most optimistic and the most pessimistic of these scenarios (2.6 W/m^2^ and 8.5 W/m^2^, respectively) to forecast the future distribution of whale sharks across the range of climate change predictions. Variables were available at a spatial resolution of 5 arc‐minutes (i.e., slightly less than 10 km^2^ in the study area).

**Table 1:** Variables used for modeling whale shark distribution obtained from bio‐ORACLE v2.0

Code	Variable	Units
BO2_tempmax_ss	Sea surface temperature (maximum)	°C
BO2_tempmean_ss	Sea surface temperature (mean)	°C
BO2_tempmin_ss	Sea surface temperature (minimum)	°C
BO2_temprange_ss	Sea surface temperature (range)*	°C
BO2_tempmax_bdmean	Seawater temperature (maximum at mean depth)	°C
BO2_tempmean_bdmean	Seawater temperature (mean at mean depth)	°C
BO2_tempmin_bdmean	Seawater temperature (minimum at mean depth)*	°C
BO2_temprange_bdmean	Seawater temperature (range at mean depth)	°C
BO2_chlomax_ss	Chlorophyll concentration (maximum)	mg/m^3^
BO2_chlomean_ss	Chlorophyll concentration (mean)	mg/m^3^
BO2_chlomin_ss	Chlorophyll concentration (minimum)*	mg/m^3^
BO2_chlorange_ss	Chlorophyll concentration (range)	mg/m^3^
BO2_curvelmax_ss	Current velocity (maximum)	m/s
BO2_curvelmean_ss	Current velocity (mean)	m/s
BO2_curvelmin_ss	Current velocity (minimum)	m/s
BO2_curvelrange_ss	Current velocity (range)	m/s
BO2_salinitymax_ss	Sea surface salinity (maximum)*	PSS
BO2_salinitymean_ss	Sea surface salinity (mean)	PSS
BO2_salinitymin_ss	Sea surface salinity (minimum)	PSS
BO2_salinityrange_ss	Sea surface salinity (range)	PSS
BO2_salinitymax_bdmean	Seawater salinity (maximum at mean depth)	PSS
BO2_salinitymean_bdmean	Seawater salinity (mean at mean depth)	PSS
BO2_salinitymin_bdmean	Seawater salinity (minimum at mean depth)	PSS
BO2_salinityrange_bdmean	Seawater salinity (range at mean depth)	PSS

Note

The asterisks indicate the variables that were selected for modeling.

To select a subset of relevant variables for modeling whale shark presence–absence, we used the *multGLM* function of the *fuzzySim* R package version 2.2 (Barbosa, 2015). This function implements a variable selection procedure that takes into account several criteria: correlations among variables (removing, from each pair of variables with an absolute correlation greater than 0.8, the one that is least informative regarding the species' occurrence); the false discovery rate (removing variables whose relationship with the species became nonsignificant after accounting for the number of variables in the dataset, hence reducing type I errors); and parsimony (performing a forward–backward stepwise selection of the remaining variables using the Akaike information criterion (AIC) until no variable provides a relevant improvement to the model). We then used the *modEvA* R package version 1.4 (Barbosa, Brown, Jiménez‐Valverde, & Real, 2016) to ensure that the selected variables provided good model calibration and discrimination performance. We also appraised the ecological relevance of these variables using related literature (e.g., Sequeira et al., 2012).

### 2.2 Species occurrence data

**Figure 1:**
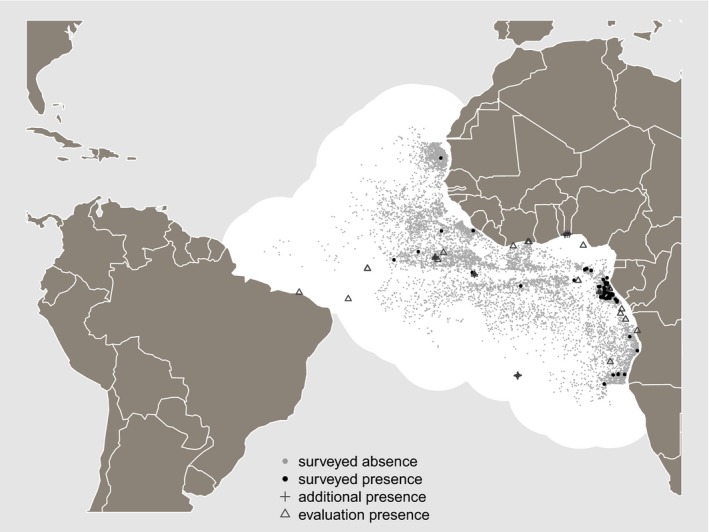
Surveyed absence and presence records, additional presences (from online sources) used for modeling whale shark distribution, and nonmodeled presences used for external evaluation. The surveyed and thus modeled area is highlighted in white

The Spanish Institute of Oceanography (IEO) observers program places one observer on each of the commercial purse seine vessels. The observers conduct a scientific program under the EU Data Collection Framework. The observers follow the same data collection and processing methodology in the Atlantic and Indian Oceans (Ariz, Chavance, Delgado de Molina, & Murua, 2010). The main aim of the observer program is to obtain direct information on bycaught species as well as the discards of target species (e.g., catch and bycatch species, number of individuals, size, and other biological data). The present study used original data recorded by the IEO from 2003 to 2016 from the above‐mentioned program. These data included 73 presence and 10,510 absence points spanning the tropical and subtropical Atlantic Ocean (Figure 1). The data reveal which of the areas encompassed by purse seine vessels have more and less presences compared to absences of whale sharks.

We used correlative models based on the observed relationships between surveyed presence/absence of whale sharks and the selected predictor variables. Areas that have not been sampled should normally be excluded from the model calibration area, and extrapolation should be avoided beyond it (e.g., Owens et al., 2013). We thus circumscribed our modeling area to a spatial buffer surrounding our surveyed points, with a radius equaling the mean pairwise great‐circle distance (calculated with the *distHaversine* function of the *geosphere* R package; Hijmans, 2017) between our presence points (Figure 1). We then complemented our dataset by including additional presence data retrieved from online public databases, which were accessed using the *spocc* R package version 0.9.0 (Chamberlain, 2018) on 4 March, 2019. Whale shark occurrence data were available from the Global Biodiversity Information Facility, iNaturalist, Bison, and Vertebrate Networks. From these data, we removed: (a) points that fell outside our spatial buffer, as they were not accompanied by surveyed absence points around the same regions and could thus mislead the models; (b) points with missing or probably incorrect coordinates (i.e., where both latitude and longitude equaled zero); and (c) points with reported location imprecision greater than 10,000 m (i.e., larger than the size of the pixels in the raster maps of the predictor variables). Following this data cleaning procedure, data from these online sources added 20 presence points to our modeling area.

To reduce both spatial bias and spatial autocorrelation in occurrence data, we performed a spatial thinning procedure by selecting only one presence or absence point within each pixel of the predictor variable maps. We first selected all pixels with at least one presence and then all remaining pixels with at least one absence point. We further eliminated 3 points which fell outside the pixels containing values for the predictor variables. The resulting dataset had 74 pixels with presence records and 8,190 pixels with absence records.

To avoid an excessive number of absences relative to the number of presences in the modeling dataset (which could cause overfitting and artificially inflate model performance metrics), while limiting the loss of relevant information and the departure from observed prevalence, we selected a random sample of the absence pixels to obtain a 1:10 presence–absence ratio in the models (Barbet‐Massin, Jiguet, Albert, & Thuiller, 2012; Sequeira, Mellin, Fordham, Meekan, & Bradshaw, 2014). Our final occurrence dataset for modeling thus comprised 74 pixels with presence and 740 pixels with absence of whale shark observations. To ensure that the random sampling of absences did not affect the selection of the most appropriate subset of predictor variables, we repeated this sampling process 1,000 times to determine the most frequently selected subset of variables and used a modeling sample that produced this selected subset.

### 2.3 Modeling

Several recent studies show that assembling models built with different algorithms are important for addressing model‐based uncertainty, particularly in the marine environment (see e.g., Robinson, Nelson, et al., 2017 for a review). We thus built a model ensemble using the selected variables, the surveyed presence and absence points, and the 19 algorithms currently implemented in the *sdm* R package version 1.0‐67 (Naimi & Araújo, 2016): Bioclim, Bioclim.dismo, Boosted Regression Trees (BRT), Classification and Regression Trees (CART), Domain, Flexible Discriminant Analysis (FDA), Generalized Additive Models (GAM), Generalized Linear Models (GLM), Lasso and Elastic‐Net Regularized Generalized Linear Models (GLMNET), Mahalanobis distance, Multivariate Adaptive Regression Splines (MARS), Maximum Entropy (MAXENT), Maxlike, Mixture Discriminant Analysis (MDA), Multi‐Layer Perceptron (MLP), Radial Basis Function (RBF), Random Forests (RF), Recursive Partitioning and Regression Trees (RPART), and Support Vector Machines (SVM). The software is open‐source and publicly available at the Comprehensive R Archive Network (https://CRAN.R-project.org/package=sdm), so the information on all modeling parameters for the different algorithms can be found there.

We built an initial set of models using 10 different runs of subsampling replications, each one reserving 15% of the data for model testing. We used these testing data for analyzing out‐of‐sample model performance (i.e., for cross‐validation), as assessed both by the area under the receiver operating characteristic curve (AUC), which measures overall discrimination capacity, and by the true skill statistic (TSS), which balances the capacity to correctly predict presences and absences. The latter is threshold‐dependent, and we used the threshold that maximized the sum of the correct classification rates of presences and absences. We excluded from further analysis algorithms with a mean test AUC under 0.7 (the minimum for model performance to be considered “fair”; Swets, 1988) or with a mean test TSS under 0.5 (considering that TSS has a larger range of variation than the AUC, that is, between −1 and 1 rather than between 0 and 1). We also excluded algorithms producing predicted values largely outside of the [0, 1] interval, which could not be accurately converted to the same scale as the other algorithms in the ensemble. Subsequently, we used the selected algorithms and the complete modeling dataset (without leaving out a test sample) to build a final model ensemble, from which we calculated the mean and variance of the predictions of the different algorithms.

Finally, we used the *predict* function of R to project the models both to current environmental conditions across the study area and to future environmental scenarios for this region. To avoid excessive extrapolation for a correlative model (Bouchet et al., 2019; Owens et al., 2013; Yates et al., 2018), we analyzed our predictions only within the surveyed region that was used for model calibration. We evaluated model performance across all pixels of the modeled region with the continuous Boyce index, implemented in the *ecospat.boyce* function of the *ecospat* R package (Broennimann, Cola, & Guisan, 2018), using a moving window with 100 focals and a width of 1/10 of the prediction range. This index measures model accuracy for presence‐only test data against a geographical‐environmental background. We calculated the Boyce index for the modeled presences, and also for an additional set of presences retrieved from the MarineSPEED dataset (Bosch, Tyberghein, Deneudt, Hernandez, & Clerck, 2018), as an external evaluation of the models.

**Figure 2:**
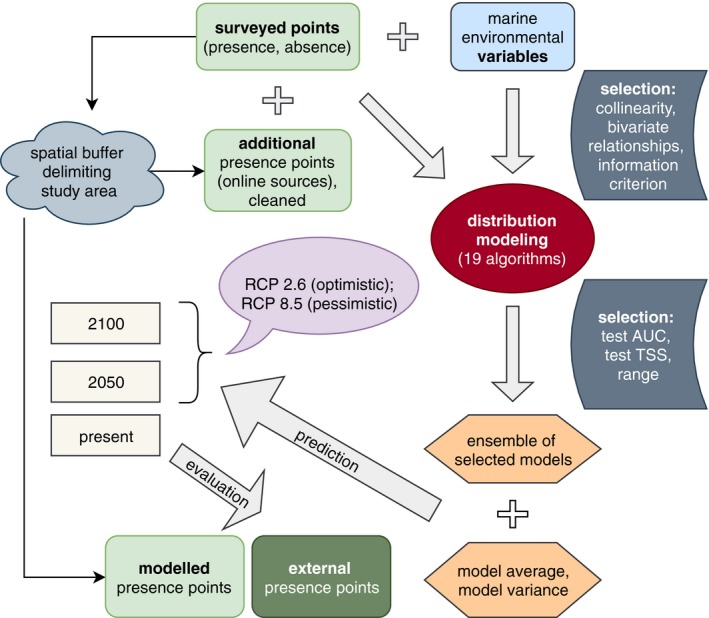
Flowchart summarizing the model building and evaluation procedure. AUC, area under the curve; RCP, relative concentration pathway; TSS, true skill statistic. For more details, please see main text

A graphical summary of our complete modeling procedure, including the selection of occurrence data and predictor variables, the selection of models, the prediction to different scenarios and the final evaluation with internal and external data, is provided in Figure 2.

## 3 RESULTS

**Figure 3:**
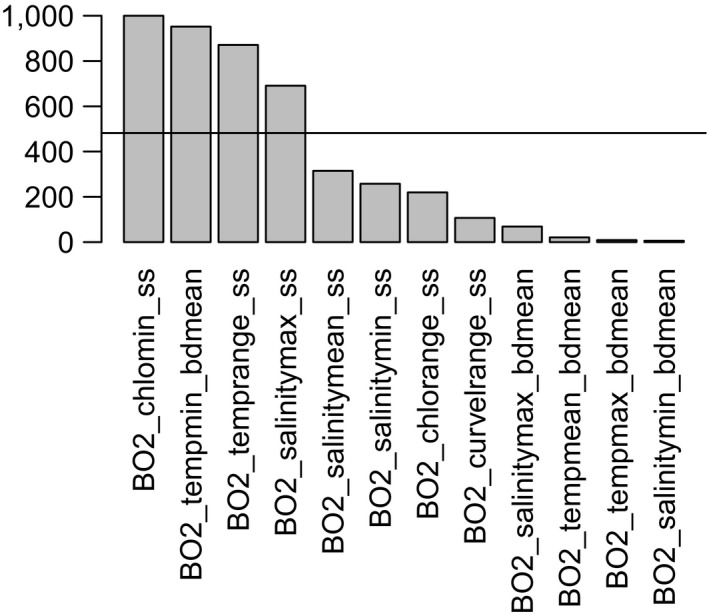
Number of times that each variable was selected using 1,000 random samples of the absence data. Variables codes as in Table 1. The horizontal line marks the number of times that the subset comprising the first four variables was chosen; all other subsets were substantially less frequent

The 1,000 random samples of absence data produced 46 unique combinations of selected variables, but with very different frequencies, and one that was clearly dominant. The most frequent combination of variables was selected 482 times, whereas the second most frequent combination (which included the same variables as the former combination plus an additional one) was selected 184 times. All the remaining combinations were selected much less frequently (Figure 3). Four variables composed the most frequently selected set: minimum seawater temperature at mean depth, sea surface temperature range, minimum sea surface chlorophyll concentration, and maximum sea surface salinity (Table 1; Figure 3). The generalized linear model obtained with these variables achieved good performance measures, with an AUC of 0.84, which is generally considered “good” (Swets, 1988), and a proportion of explained deviance (*D* ^2^) and McFadden's pseudo‐*R* ^2^ of .23, which is considered “excellent fit” (McFadden, 1978). We also confirmed that these variables were ecologically meaningful given existing literature on the target species (see Section 4).

**Figure 4:**
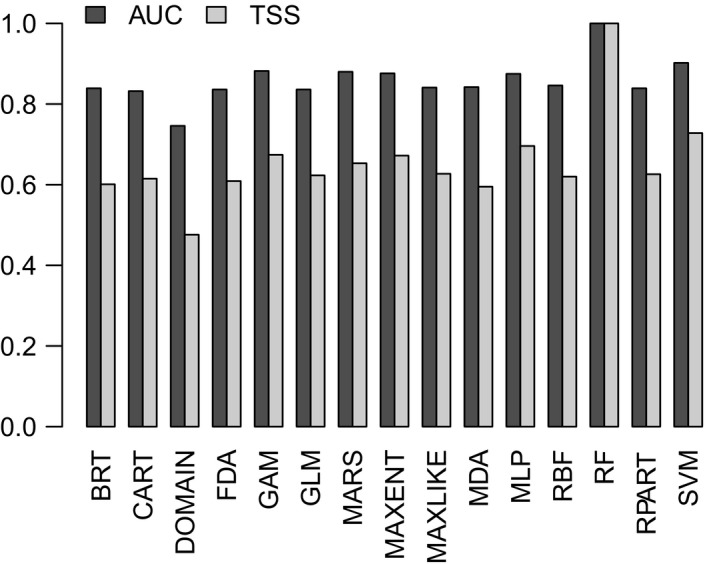
Discrimination performance of the algorithms included in the model ensemble, as measured by the area under the receiver operating characteristic curve (AUC) and the true skill statistic (TSS). Please see main the text for model algorithm abbreviations

Using the selected subset of variables, 15 modeling algorithms met the performance thresholds and were included in the final model ensemble: BRT, CART, DOMAIN, FDA, GAM, GLM, MARS, MAXENT, MAXLIKE, MDA, MLP, RBF, RF, RPART, and SVM. All algorithms showed good discrimination performance on the modeled dataset (Figure 4). Variable importance plots for each algorithm are represented in Appendix S1 (Figure S1).

**Figure 5:**
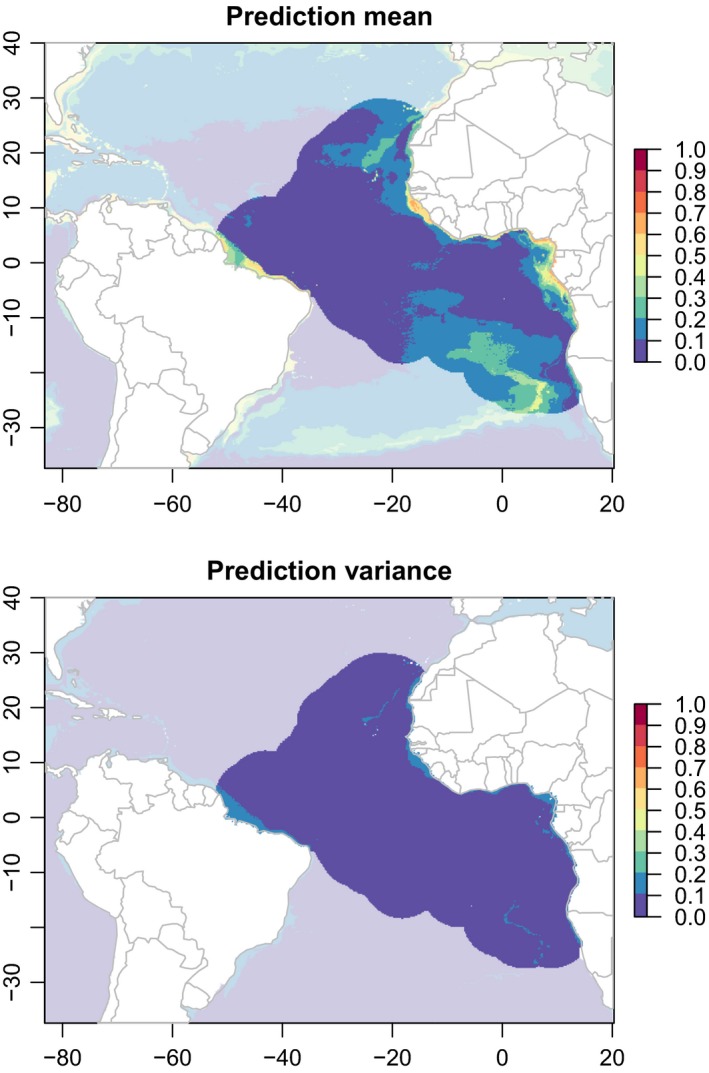
Mean and variance of environmental suitability predictions by the ensemble model algorithms for current whale shark occurrence in the Atlantic Ocean. Predictions outside the modeled region are faded out. The predictions of each individual algorithm are shown in Appendix S1

According to the results, the current suitable habitats for whale sharks cover an area larger than current observations suggest, although some regions show greater prediction variance among modeling algorithms (Figure 5). Across all pixels of the modeling region defined by our spatial buffer, the Boyce index of the averaged model predictions was 0.83 for the modeled presences and 0.87 for the external evaluation presences.

**Figure 6:**
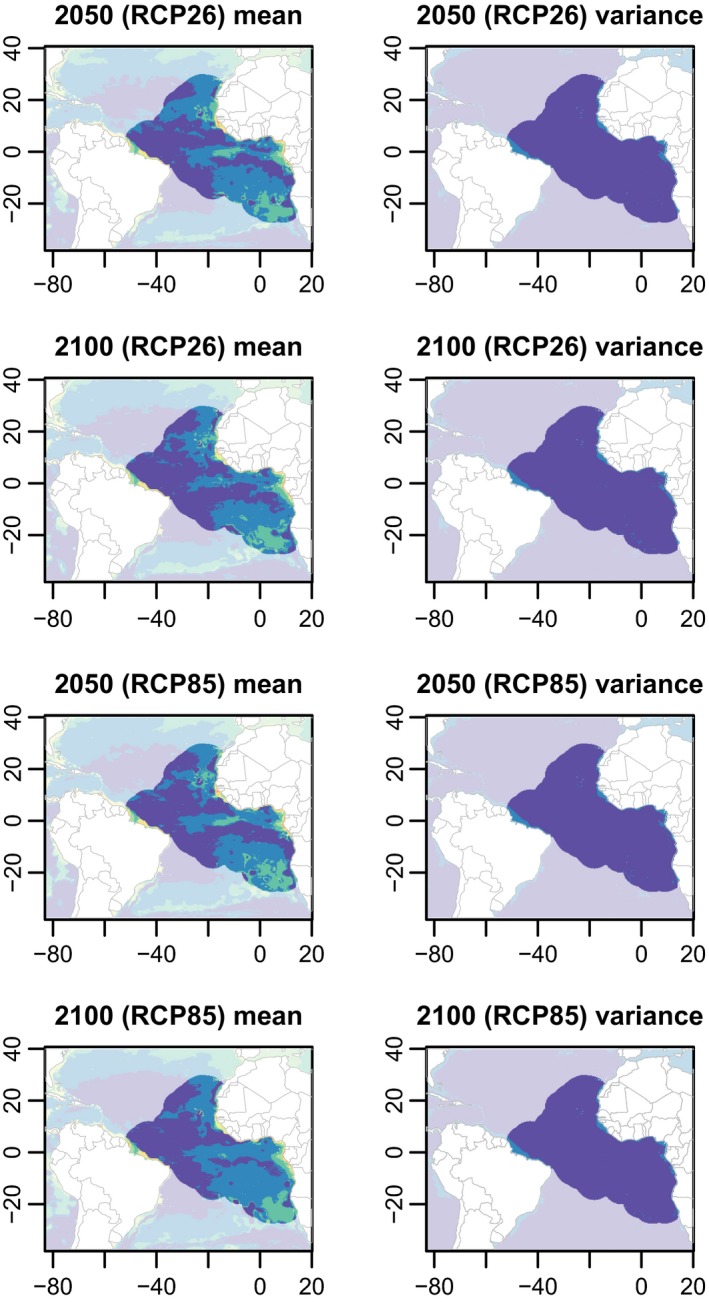
Mean and variance of environmental suitability predictions by the ensemble model algorithms under future projections for 2040–2050 and 2090–2100, including two greenhouse gas concentration scenarios based on different representative concentration pathways (RCP). Predictions outside the modeled region are faded out. The predictions of each individual algorithm are shown in Appendix S1

When applied to future projections for the modeled region, these models predicted that the suitable environmental conditions for this cosmopolitan oceanic species could expand to higher latitudes (Figure 6).

## 4 DISCUSSION

Although the whale shark is a charismatic species, little is known about its global distribution (Sequeira et al., 2013). Distribution modeling studies are thus essential for optimizing the available information on occurrence sites and their environmental traits, in order to predict the suitable areas for the current and future occurrence of this species (Robinson, Nelson, et al., 2017). In this study, we presented a model ensemble of a newly collected dataset on whale shark occurrence in the Atlantic Ocean, endeavoring to follow current best‐practice recommendations in the field.

Most of the variables selected for our models were congruent with existing literature on the target species, as predictors related to water temperature and chlorophyll concentration were also previously found relevant in the Atlantic and Indian Oceans (Escalle et al., 2016; Sequeira et al., 2012, 2014). Sea surface temperature (SST), in particular, is a relevant environmental variable linked to whale shark global distribution (Sequeira et al., 2013). Thus, according to Sequeira et al. (2012), 90% of sightings recorded in the Indian Ocean occurred with SSTs between 26.5°C and 30°C. Sequeira et al. (2012) hypothesized that whale sharks might avoid high or low temperatures, which could increase their metabolic rates or limit their metabolic function, respectively. Previous studies on the potential effect of climate change on whale shark distribution have suggested that warming SSTs could lead to a redistribution of the species as a function of this variable (Sequeira et al., 2014). Thus, predicted changes in sea surface temperature resulted in a slight shift of suitable habitat toward the poles in both the Atlantic and Indian Oceans (Sequeira et al., 2014), which is in agreement with the results of this paper. The current key aggregation areas for whale shark in the Atlantic Ocean are located north of the Gulf of Mexico, off the Yucatan Peninsula, and off Belize (Copping et al., 2018). The seas around the São Pedro and São Paulo archipelago are also relevant to this species (Macena & Hazin, 2016), given their large concentrations in this area. Currently, the biological significance of these aggregations is not well understood (Sequeira et al., 2013). On the other hand, adult whale sharks spend most of their lives in deep offshore waters, which are thought to be predominantly seasonal habitats (Abercrombie, Balchowsky, & Paine, 2005; Acuña‐Marrero et al., 2014; Andrzejaczek et al., 2016). In this context, the present results only apply to deep offshore habitats (i.e., the areas where most of the sightings of this species occur over the year). Thus, we predict part of the habitat and population structure of the whale shark and suggest which areas near the ocean surface are suitable for presences.

On a coarser spatial scale and using count data for the Eastern Atlantic Ocean, Escalle et al. (2016) also found the coastal waters of west‐central Africa, including Angola, Congo, Equatorial Guinea and especially Gabon, to be areas of high potential co‐occurrence of whale sharks and purse seine fisheries. Similar predictions were obtained by Sequeira et al. (2014) with presence‐pseudoabsence data and generalized linear mixed models. Currently, the area off the coast of Gabon is a relevant feeding site for whale sharks (Escalle et al., 2016). Under existing climate projections, this key area, as well as the São Pedro and São Paulo archipelago (Brazil), is also predicted as relevant future sites by our models. These findings provide support to our methods and results, as our data did not include presences in these regions, although their importance is documented in the literature (Macena & Hazin, 2016). For this reason, the methodology used in the present study could be a useful tool to model other vulnerable marine species.

Some areas with existing presence points were predicted as having low suitability by our models; but these areas also showed many more absence than presence points. We remark that we modeled presences against surveyed absences, rather than randomly spreading background or pseudoabsence points throughout the modeled region. While absence is always uncertain even where observers were active, a high number of surveyed absences in an area indicate that the area is not so frequently visited by the target species. Areas with only occasional presences and low predicted suitability may thus indicate vagrant individuals or dispersal paths.

Providing that the current key suitable areas are maintained, in the future they could be considered as refugia. In this sense, many authors have suggested that whale sharks display strong site fidelity to concentration areas (e.g., McCoy et al., 2018; Sequeira et al., 2013). Although a priori these areas could help mitigate the impacts of climate change on the species, they might also become ecological traps, as they could lose suitability due to decreased future productivity.

On the other hand, our predictions suggest that the distribution of suitable areas may slightly shift toward the north (between Sierra Leone, Liberia, and the Ivory Coast) and the south (the Gulf of Benguela in Angola) and that this distribution will undergo major dispersion. The predictions also suggest that the Gulf of Gabon will not be an area of special concentration in the future (Figures 4 and 5). Suitable areas could play an important role in the future survival of this species.

Our models do not predict a strong reduction in suitable areas for whale shark in the Atlantic Ocean in the next decades, even under the most pessimistic greenhouse gas concentration pathway. However, climate change could have negative impacts on the biology and management of the whale shark. The dispersion of suitable future areas implies that zooplankton might not have available the nutrients needed for blooms. Currently, zooplankton blooms are strongly associated with specific areas due to upwelling systems, currents, estuary rivers, or other topographic conditions that increase nutrients in the area. However, shifts in suitable areas for whale shark do not always imply that new zooplankton blooms will occur in these areas. These shifts could be another ecological trap for the whale shark.

The changes predicted by current projections refer to a relatively short time period (i.e., 80 years). Given that whale sharks have a long life span and reach maturity late, they could find it difficult to adapt to the new environmental conditions. Moreover, whale shark mating, pupping, and foraging grounds can cover thousands of kilometers in both pelagic and shallow waters, which would make their conservation difficult in the context of climate change.

Finally, although the whale shark is a planktivorous elasmobranch, it also feeds on coral spawn, and so aggregations of whale sharks near Australia could be associated with coral spawn episodes (Taylor, 1996). Coral reef regression primarily caused by climate change is one of the multiple threats that could affect the future global distribution of whale sharks, and thus also their future distribution in the Atlantic Ocean, although these additional threats could not be taken into account in this study. Further studies are needed to predict key pelagic and shallow water areas for the whale shark involving its entire population structure. One of the limitations of the present study is that the observed distribution of whale shark is seasonally dependent (e.g., McCoy et al., 2018; Sequeira et al., 2013), whereas we used annual mean values for the analyzed variables, providing a broad‐scale prediction of generally suitable areas. Further studies using occurrence datasets containing sufficient presences for each season, as well as seasonal future projections for environmental variables, may allow season‐based predictive models to provide a more thorough overview of whale shark distribution.

## CONFLICT OF INTEREST

All authors declare they have no conflict of interest.

## AUTHOR CONTRIBUTIONS

JCB and AMB conceived the ideas and designed methodology; MLR, PP, and FA collected the data; AMB analyzed the data; JCB wrote a first draft of the manuscript. All authors contributed critically to the drafts and gave final approval for publication.

## Data Availability

Data are available on Dryad: https://doi.org/10.5061/dryad.rfj6q576m.
