# Supplementary material for: Ensemble modeling of the potential distribution of the whale shark in the Atlantic Ocean
Source: Ecol Evol. 2019 Nov 28;10(1):175–84. doi: 10.1002/ece3.5884 (PMC6972796; doi:10.1002/ece3.5884)
Supplement: Supplementary file 1 [file ECE3-10-175-s001.pdf]

SUPPLEMENTARY INFORMATION

to the article

Ensemble modelling of the potential distribution of the whale shark in the Atlantic Ocean

José C. Báez[1,2,*], A. Márcia Barbosa[3], Pedro Pascual[4], M. Lourdes Ramos[4], Francisco Abascal[4]

*Corresponding author: granbaez_29@hotmail.com; josecarlos.baez@ieo.es

[1] Instituto Español de Oceanografía, Centro Oceanográfico de Málaga, Fuengirola (Málaga), Spain.
[2] Universidad Autónoma de Chile, Facultad de Ciencias de la Salud, Santiago de Chile, Chile.
[3] Universidade do Porto, Faculdade de Ciências, CICGE - Centro de Investigação em Ciências Geo-Espaciais, Observatório Astronómico Prof. Manuel de Barros, Alameda do Monte da Virgem, 4430-146 Vila Nova de Gaia, Portugal.
[4] Instituto Español de Oceanografía, Centro Oceanográfico de Canarias, Santa Cruz de Tenerife, Spain.

This document contains 6 supplementary figures, including variable importance plots and environmental suitability prediction maps for each of the 15 algorithms in the ensemble of models of whale shark distribution in the Atlantic.

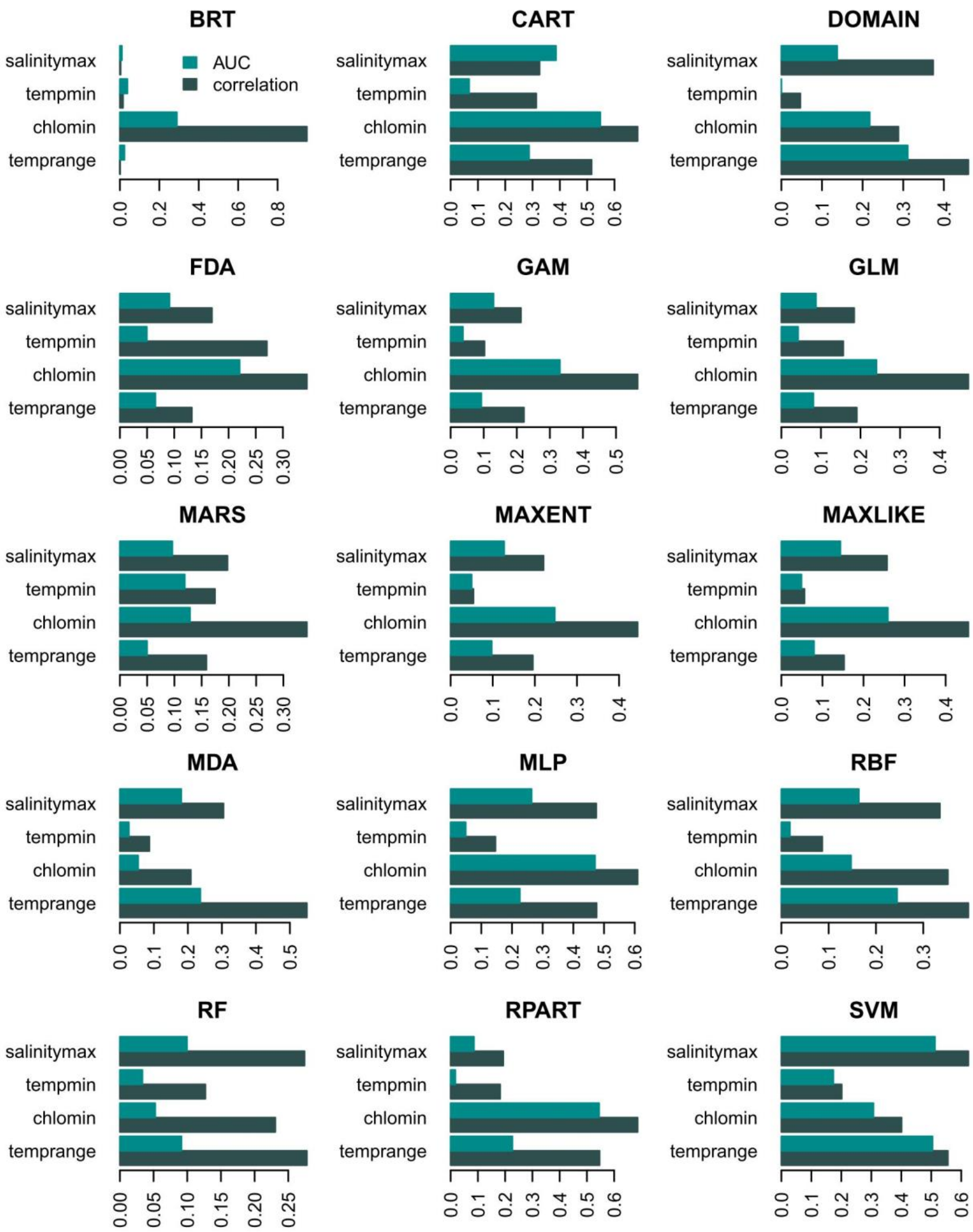

Figure S1. Variable importance plots, according to the AUC test and the correlation test, for each of the 15 algorithms and 4 variables selected for the model ensemble. Values were obtained with the *sdm* R package. Please see the main text for the meanings of the algorithm abbreviations, and Table 1 in the main text for a description and source of the variables.

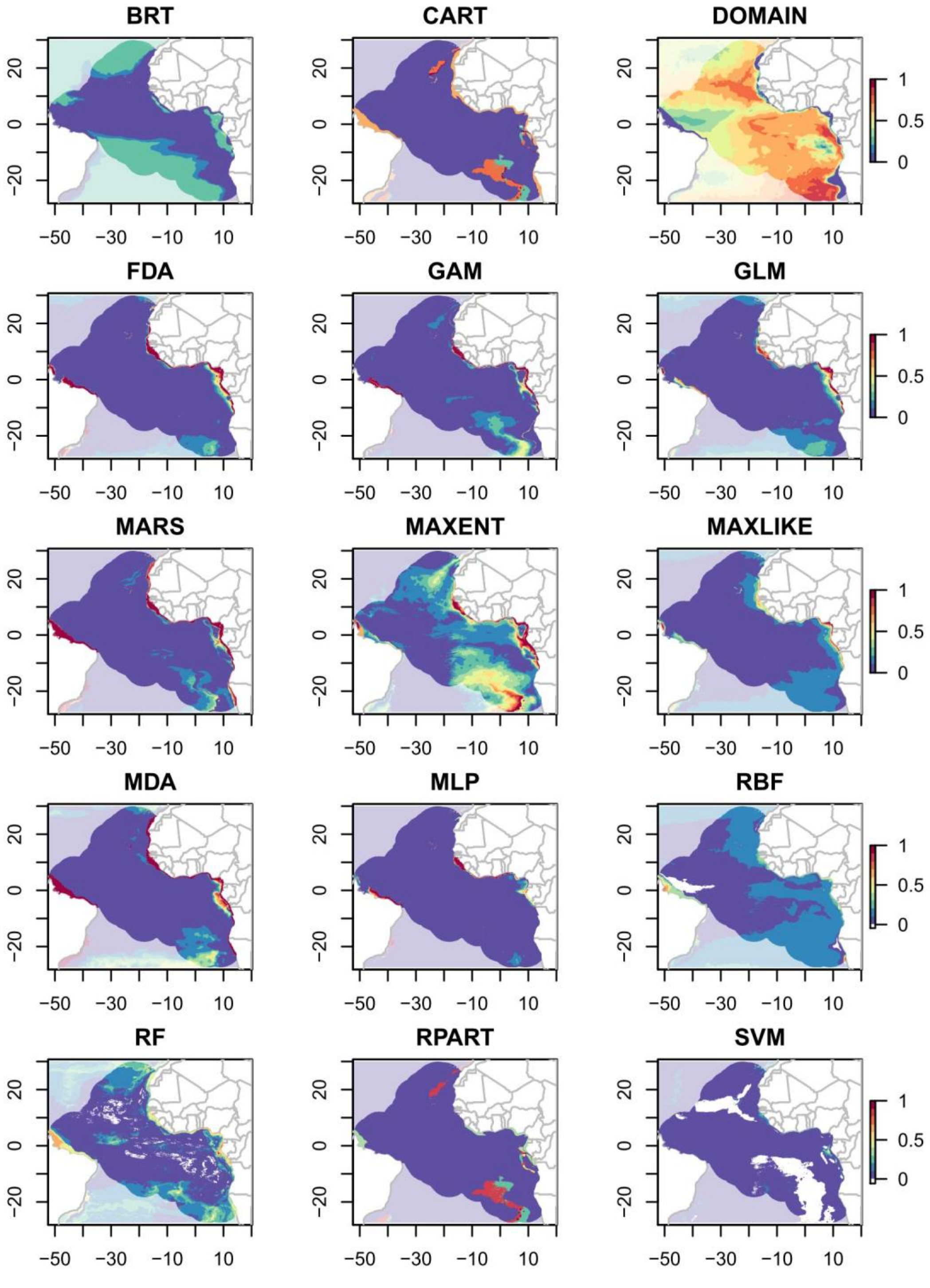

Figure S2. Environmental suitability predictions by each algorithm in the ensemble of models of current whale shark distribution in the Atlantic Ocean. Please see the main text for algorithm abbreviations.

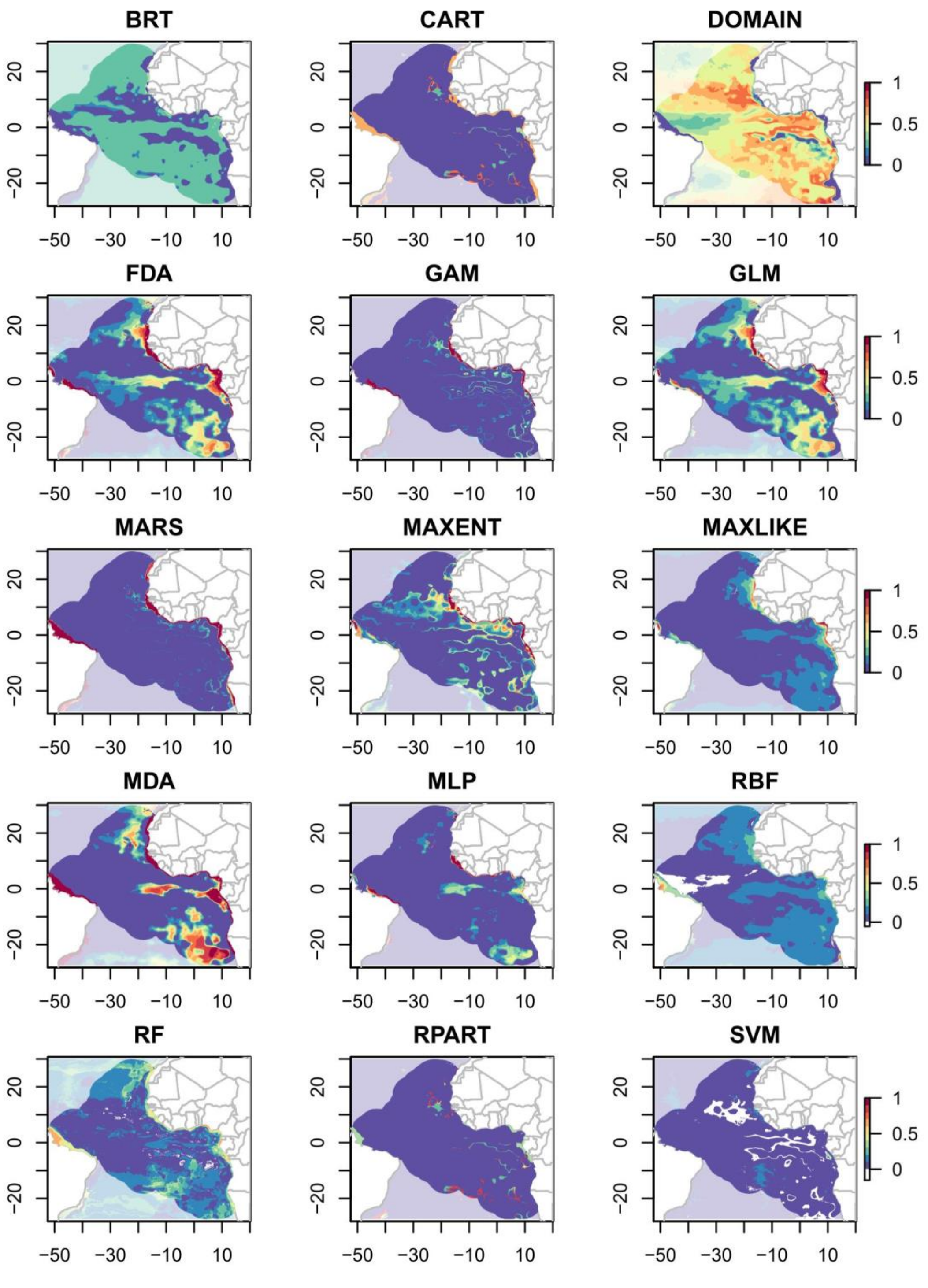

Figure S3. Environmental suitability predictions by each algorithm in the ensemble of models of whale shark distribution for 2040 to 2050, under a representative concentration pathway of 2.6 W/m^2. Please see the main text for algorithm abbreviations.

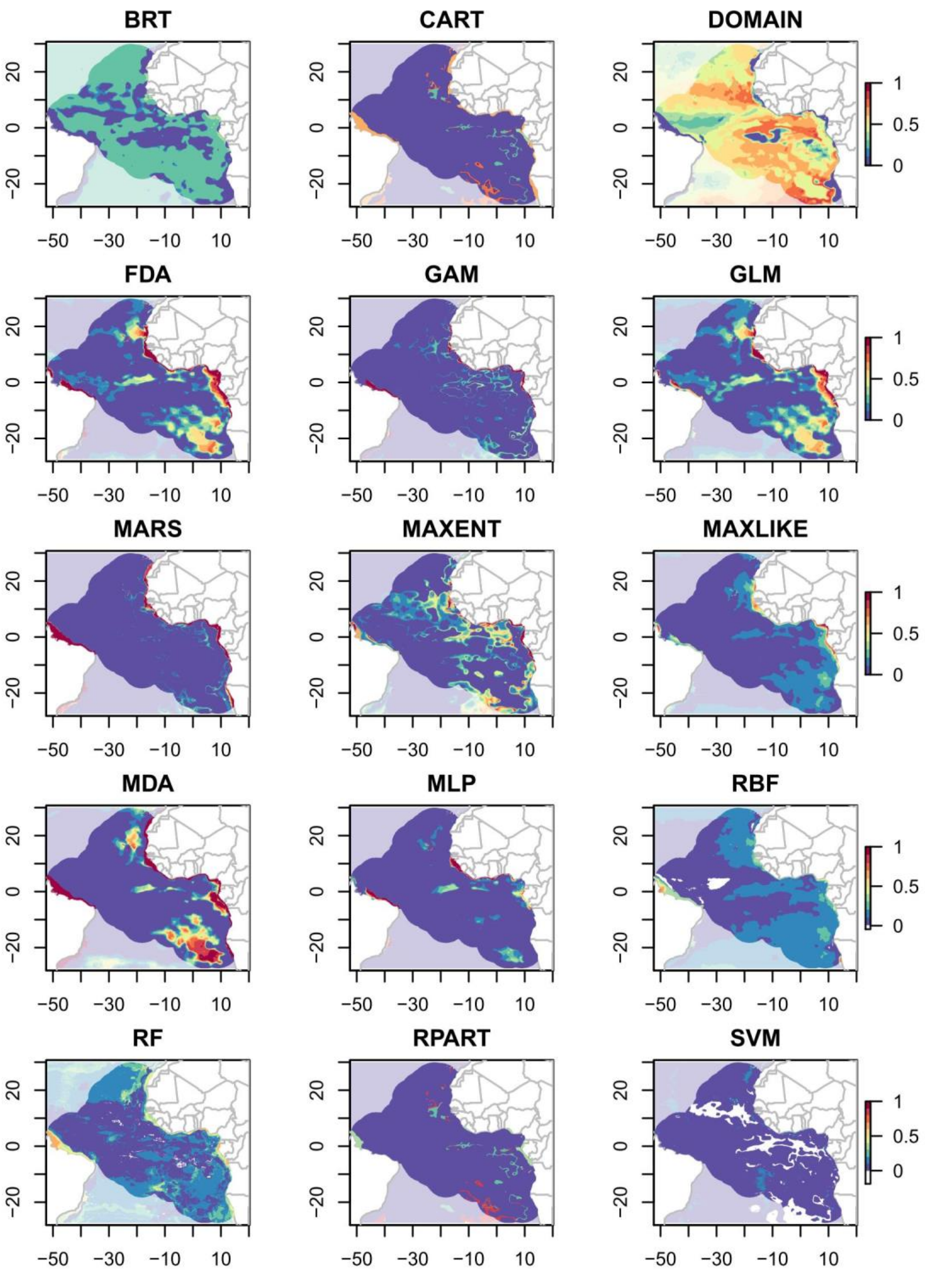

Figure S4. Environmental suitability predictions by each algorithm in the ensemble of models of whale shark distribution for 2090 to 2100, under a representative concentration pathway of 2.6 W/m^2. Please see the main text for algorithm abbreviations.

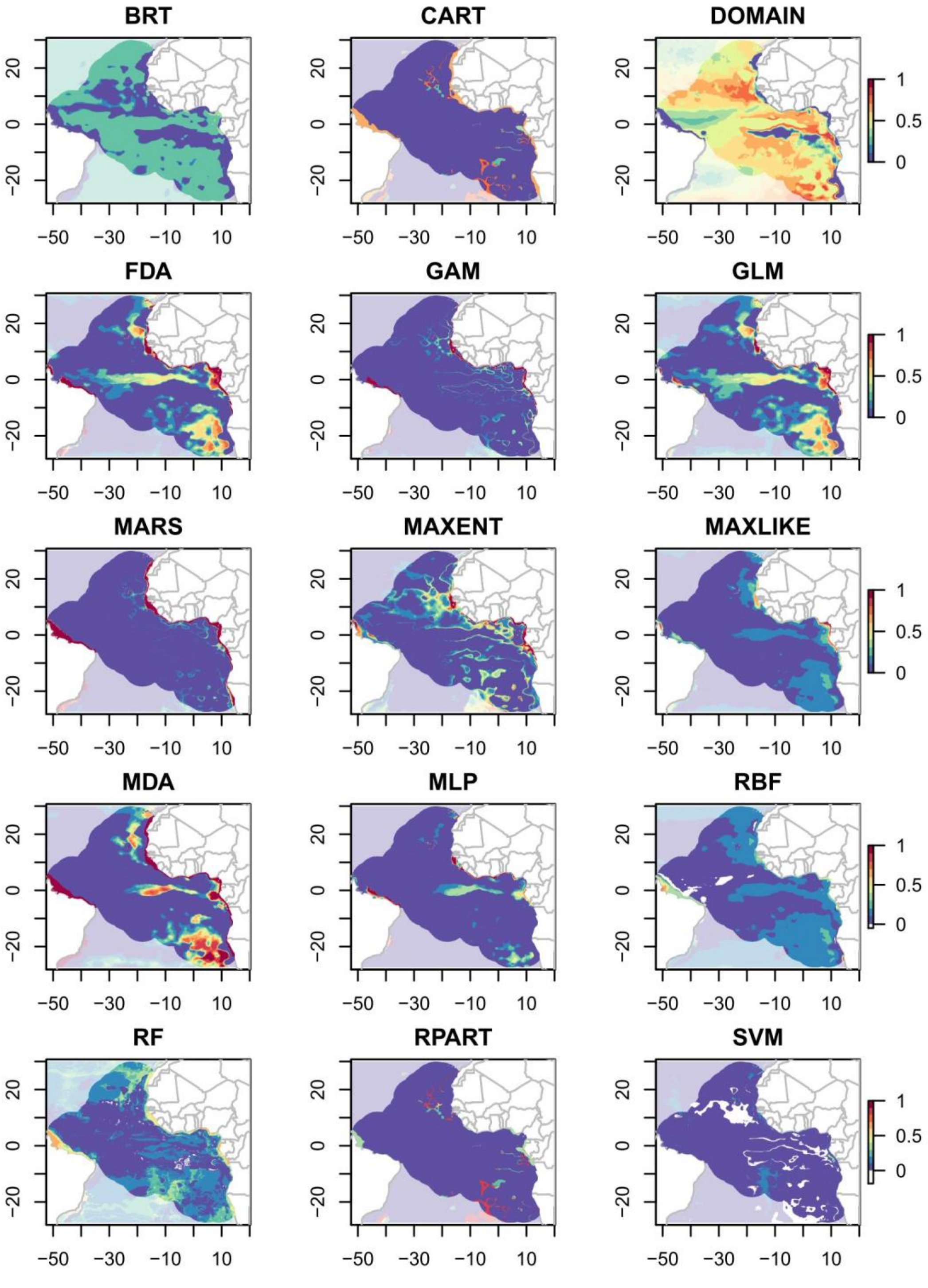

Figure S5. Environmental suitability predictions by each algorithm in the ensemble of models of whale shark distribution for 2040 to 2050, under a representative concentration pathway of 8.5 W/m^2. Please see the main text for algorithm abbreviations.

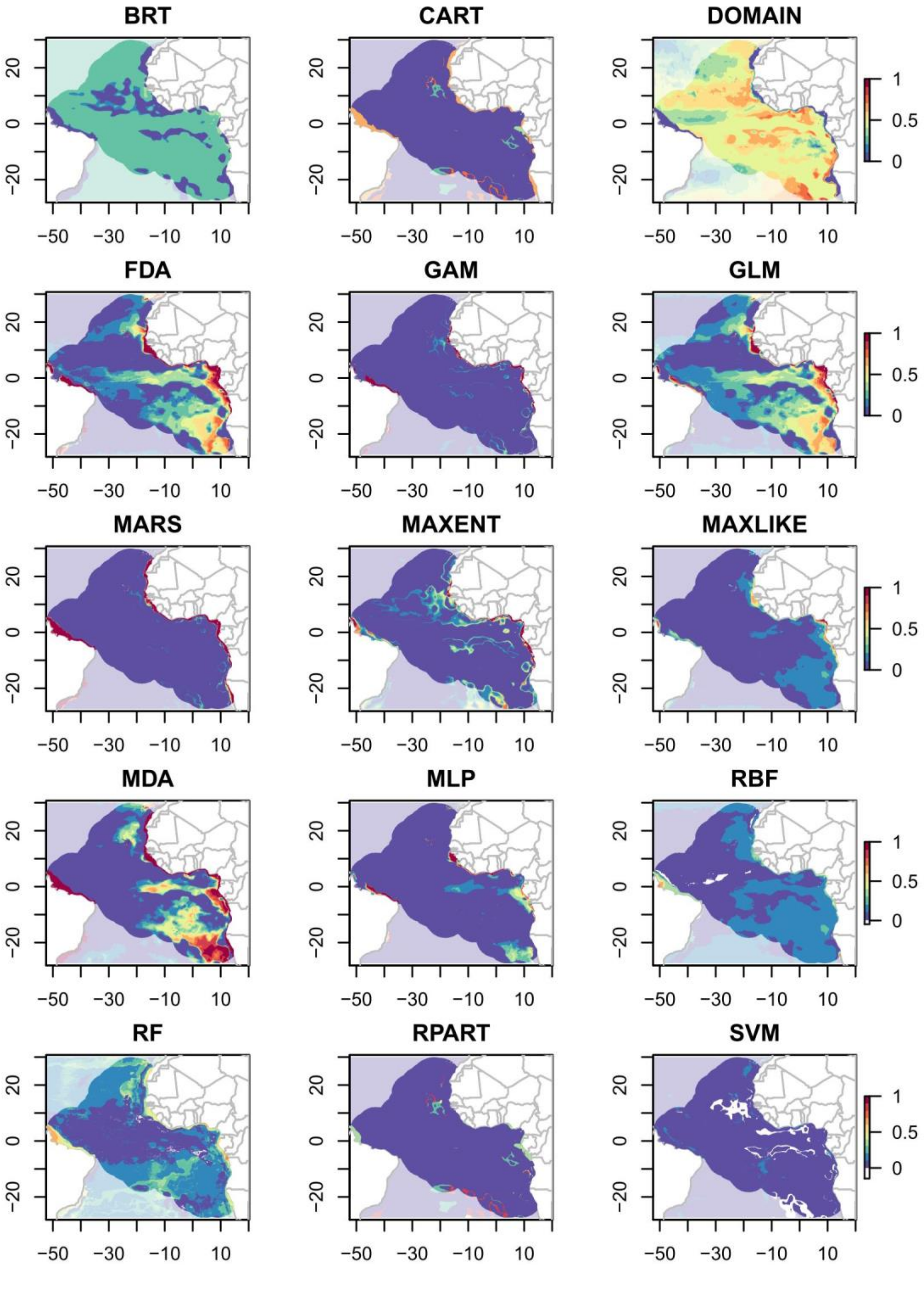

Figure S6. Environmental suitability predictions by each algorithm in the ensemble of models of whale shark distribution for 2090 to 2100, under a representative concentration pathway of 8.5 W/m^2. Please see the main text for algorithm abbreviations.
